# Deviated nose correction by using the spreader graft in the convex side

**DOI:** 10.1016/S1808-8694(15)31042-9

**Published:** 2015-10-19

**Authors:** Pedro Wey Barbosa de Oliveira, Rogério Pezato, Luiz Carlos Gregório

**Affiliations:** 1 MD. 1st Year Resident - Paulista School of Medicine - UNIFESP.; 2 M.S. in Plastic Surgery - Paulista School of Medicine - UNIFESP, Otorhinolaryngologist.; 3 PhD in Medicine (Otorhinolaryngology) - Paulista School of Medicine - UNIFESP. Head of the Otorhinolaryngology Discipline - Paulista School of Medicine - UNIFESP. Study conducted by the Discipline of Otorhinolaryngology - Paulista School of Medicine - Federal University of São Paulo. Pirajussara General Hospital.

**Keywords:** graft, nose, rhinoplasty

## Abstract

A deviated nose is the result of a set of anatomical abnormalities, and for this reason there are many methods for correcting such defects. Therefore we should not use only one single method in all cases of nasal deformities. **Aim:** In this prospective study we propose a new method using a spreader graft on the convex side of the deviated nose. **Methods:** We performed rhinoplasty in six patients by inserting a spreader graft in the convex side of the deviated nose and followed them for two years. **Results:** All six patients presented an improvement in the external appearance of their noses. Conclusions: This study showed that in some particular cases, the spreader graft technique can be used successfully to correct deviated noses.

## INTRODUCTION

Deviated noses represent the most challenging problems in rhinoplasty^1^, ^2^, ^3^, ^4^. It is particularly challenging because it comes associated with an airflow alteration in the nasal cavity; and moreover, there are usually numerous anatomical alterations,^1^ such as nasal septum cartilage alterations and that of its lateral portion, misalignment of the septal cartilage on the maxillary bone, nasal tip rotation, nasal bone asymmetry and deformities. Deviated noses are often related to nasal trauma^5^, ^6^.

If the variables that contribute to nasal deviation are not properly identified, there is the possibility of surgical treatment faillure^4^. Another cause of nasal deviation recurrence is the propensity to nasal pyramid migration towards its original position, and it may happen months after rhinoplasty^7^.

There are a number of ways to call a deviated nose: deflected nose, twisted nose, scoliotic nose, shifted nose. Its classification depends on the portion of the nose that is deviated, whether it is involving bone tissue, cartilage or both, and the anatomical structures involved.

The surgical treatment may consist on “defect camouflage”^8^, ^3^ or through anatomical reconstruction^2^.

Some authors use the spreader graft (a graft made up from the nasal septum cartilage from the patient him/herself) in order to unilaterally correct the nasal deviation on the deviation concave surface^2^. In this study, we propose the unilateral use of a spreader graft on the side to which the nasal tip is pointing, where most of the time is the convex portion of the nose.

## MATERIALS AND METHODS

### Sample

From May of 2003 through April of 2004, six patients with nasal deviation from the Department of Otorhinolaryngology underwent a rhinoseptoplasty procedure. Four were females and two were males. Of these, only one had a history of trauma prior to the nasal deviation. All the patients were followed up for a period of two years. Protocol number 0899/06 at the UNIFESP-EPM - Research Ethics Committee.

### Operative Assessment

#### Photographies

The patients were photographed before surgery, six months and two years after the procedures, frontal view, side view and caudal-cranial.

### Operative technique

Patients in dorsal decubitus underwent general anesthesia with assisted ventilation. We made V-shaped incisions on the nasal columella inferior third, encompassing skin and subcutaneous tissue, extending it to the mucous portion of the marginal nasal cavity and caudal portion of the alar cartilage. Through this incision we exposed the alar cartilages. We dissected bellow the pericondrium and periosteum, exposing the nasal dorsum together with the cartilaginous septum and its lateral projections.

After the bilateral submucosal-perichondrial dissection of the cartilaginous septum we resected its deviated portion and separated its lateral portions. Following that, the cartilaginous septum was detached from its bony portion and from the maxillary bone, in order to be repositioned on the maxilla middle line.

After septum repositioning, we resected the cartilaginous dorsum excess and placed a spreader graft (a cartilage bar) taken from the septoplasty between the septal cartilage and its lateral projection only on the nose convex deviation side. After that, we used a U-shaped suture to join the septum lateral projections to the spreader graft and the remaining nasal septum.

In all the patients, we removed the cranial portion of the alar cartilage, preserving its mucosal lining, and rebuilt the nasal tip with suturing. When necessary, we carried out turbinectomy, bony resection of the nasal dorsum or the bilateral osteotomy of the maxilla frontal process (lateral osteotomy). Skin and mucosa were sutured and the nasal cavity was packed.

## RESULTS

We obtained cosmetic improvement in all the patients (see photographies).

## DISCUSSION

The most important step in nasal deviations is to pin-point causes and correct them. There is no doubt about the impact brought about by the spreader graft on middle valve expansion^9^. Some authors use the spreader graft unilaterally, on the concave nose side in order to correct the deviated nose^2^, ^10^, since most of the times this side presents a narrower middle valve when compared to its convex counterpart.

It is extremely hard to classify the deviate nose, because it is not always that we have a concave and a convex side. The classical C-Shaped or S-Shaped, for nature’s own reasons, may have both the radix and the nasal tip located in the middle line, and in these patients this represents more of a nasal asymmetry than a true nasal deviation.

On the deviated nose, the septal cartilage may be twisted not only on the sagittal or frontal planes, but also along the nasal floor^11^. It is fundamental to locate the nasal deviation cause and correct it individually, and not only use some generalized formula^5^. According to this principle, on the present study we found one single anatomical particularity that allowed us to correct the nasal deviation using the spreader graft on one side of the deviation, coincidently on the convex side -whenever it was possible to classify them into concave and convex. These patients had their septums inserted outside of the maxilla crest, septal cartilage angle, lateral cartilage of different sizes, and nasal deviation contralateral to the lower septum insertion (Figure 1).

**Patient Number 1:**
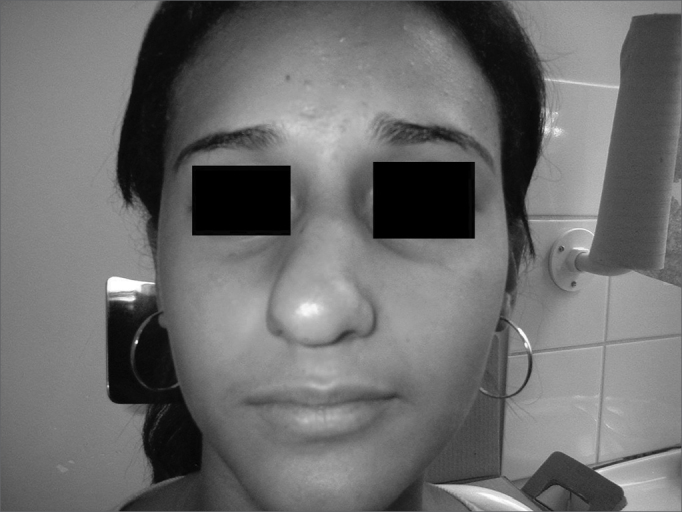
Pre-op

**Patient Number 1:**
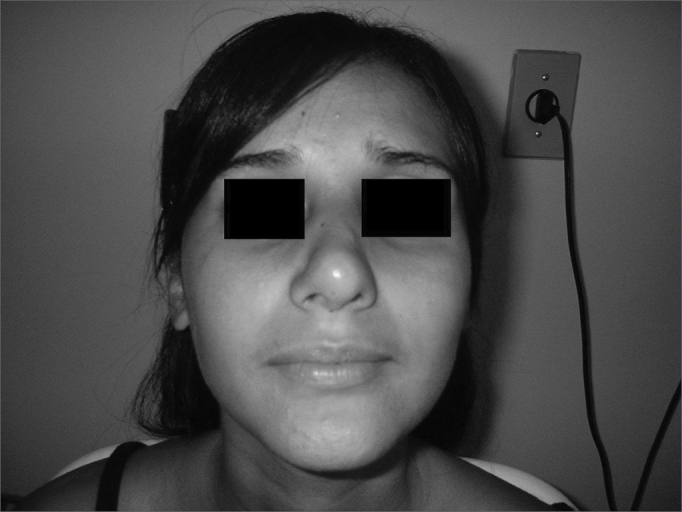
Post-op.

**Patient Number 2:**
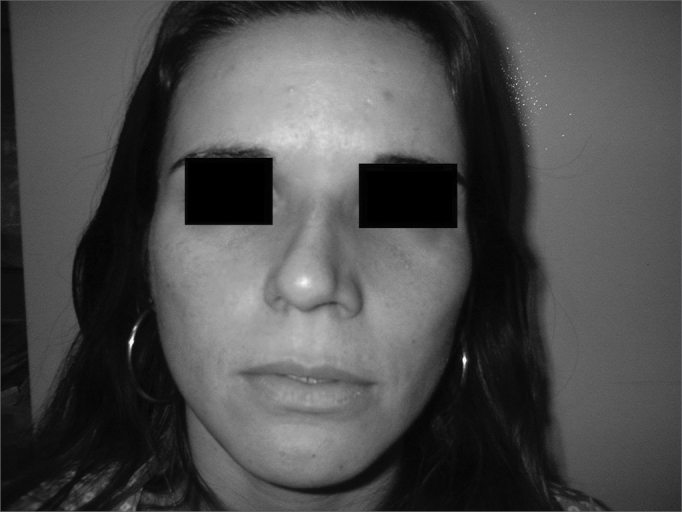
Pre-op

**Patient Number 2:**
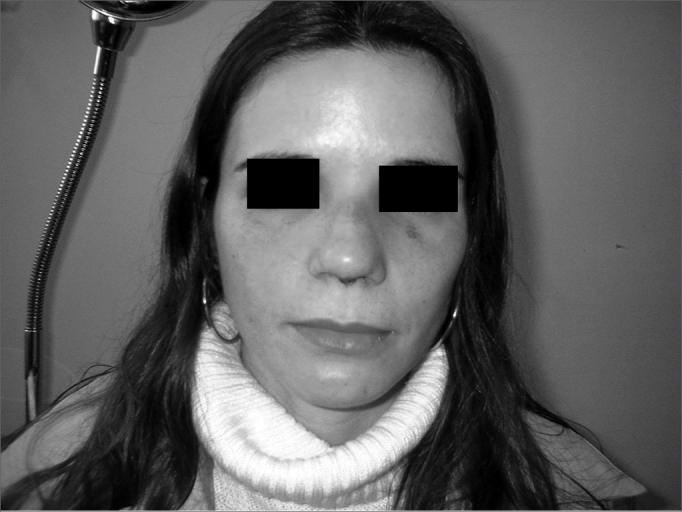
Post-op.

**Patient Number 3:**
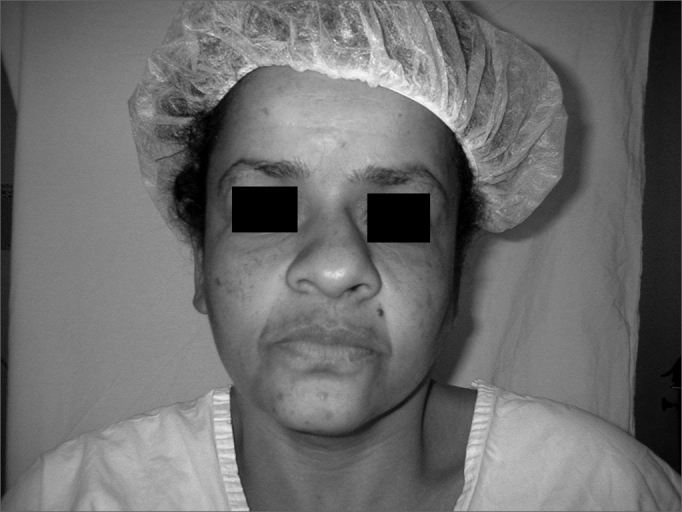
Pre-op

**Patient Number 3:**
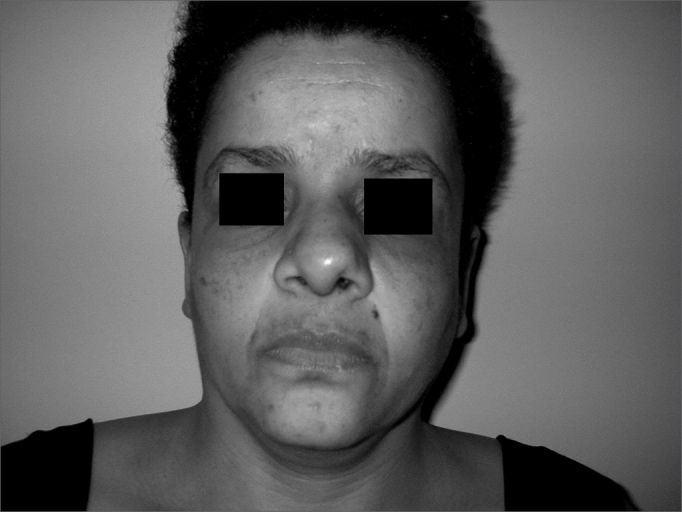
Post-op.

**Patient Number 4:**
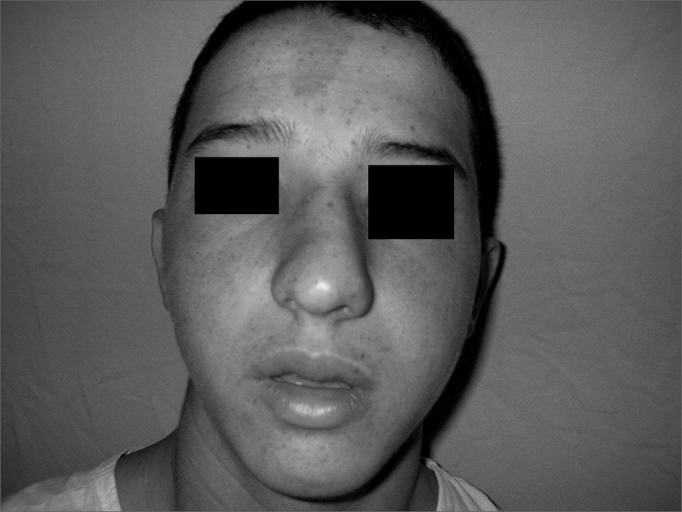
Pre-op

**Patient Number 4:**
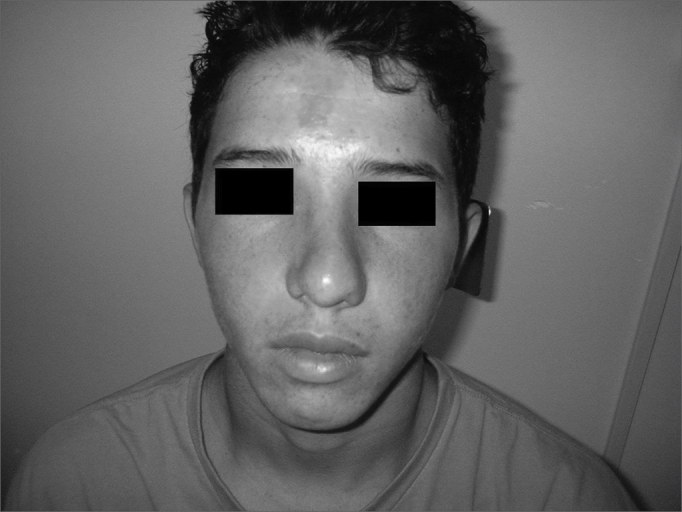
Post-op.

The spreader graft was placed on the convex side, since the septal portion that makes up the middle valve is deviated towards the convex side, and moreover, should the spreader graft be placed on the concave side for alignment and superior septum restraining purposes, with its weight it could help lead the septum back to its original position, away from the maxillary crest.

This study aimed at evaluating and justifying the possibility of unilaterally using the spreader graft on the convex portion of the nasal deviation, with a relative success reached with the aforementioned patients, and investigate the function of the unilateral spreader graft used to correct the deviated nose.

## CONCLUSION

This investigation showed that it is possible to use the spreader graft on the convex side of the nasal deviation in cases of specific anatomical alterations.
